# The Use of a Kinetic Therapy Rotational Bed in Pediatric Acute Respiratory Distress Syndrome: A Case Series

**DOI:** 10.3390/children7120303

**Published:** 2020-12-17

**Authors:** Daniel T. Cater, Aimee R. Ealy, Erin Kramer, Samer Abu-Sultaneh, Courtney M. Rowan

**Affiliations:** 1 Department of Pediatrics, Division of Critical Care, Indiana University School of Medicine, Indianapolis, IN 46202, USA; sultaneh@iu.edu (S.A.-S.); coujohns@iu.edu (C.M.R.); 2 Riley Hospital for Children, Pediatric Critical Care, Indiana University Health, Indianapolis, IN 46202, USA; AEaly@IUHealth.org (A.R.E.); ekramer@iuhealth.org (E.K.)

**Keywords:** acute respiratory distress syndrome, prone position, Intensive care units, pediatrics, critical illness, artificial respiration

## Abstract

Patients with acute respiratory distress syndrome (ARDS) commonly have dependent atelectasis and heterogeneous lung disease. Due to the heterogenous lung volumes seen, the application of positive end expiratory pressure (PEEP) can have both beneficial and deleterious effects. Alternating supine and prone positioning may be beneficial in ARDS by providing more homogenous distribution of PEEP and decreasing intrapulmonary shunt. In pediatrics, the pediatric acute lung injury and consensus conference (PALICC) recommended to consider it in severe pediatric ARDS (PARDS). Manually prone positioning patients can be burdensome in larger patients. In adults, the use of rotational beds has eased care of these patients. There is little published data about rotational bed therapy in children. Therefore, we sought to describe the use of a rotational bed in children with PARDS. We performed a retrospective case series of children who utilized a rotational bed as an adjunctive therapy for their PARDS. Patient data were collected and analyzed. Descriptive statistical analyses were performed and reported. Oxygenation indices (OI) pre- and post-prone positioning were analyzed. Twelve patients with PARDS were treated with a rotational bed with minimal adverse events. There were no complications noted. Three patients had malfunctioning of their arterial line while on the rotational bed. Oxygenation indices improved over time in 11 of the 12 patients included in the study while on the rotational bed. Rotational beds can be safely utilized in pediatric patients. In larger children with PARDS, where it may be more difficult to perform a manual prone position, use of a rotational bed can be considered a safe alternative.

## 1. Introduction

Patients with acute respiratory distress syndrome (ARDS) commonly have large zones of dependent atelectasis that contribute to intrapulmonary shunt [1,2]. The utilization of positive end expiratory pressure (PEEP) has been demonstrated to improve intrapulmonary shunt and oxygenation [3]. However, due to the heterogenous nature of ARDS, the application of PEEP can cause both recruitment of dependent atelectatic lung and over-distension of other lung regions [4]. Alternating supine and prone positioning may be beneficial to improve ventilation/perfusion matching and provide more homogeneous distribution of positive end expiratory pressure (PEEP) [5,6,7]. A meta-analysis showed that prone positioning was useful, especially in patients with severe ARDS [8]. In a large randomized controlled trial of 466 adults with ARDS by Guérin et al., early prone positioning improved survival [9]. Recently, prone positioning has also gained popularity as an adjunctive treatment modality for patients with Coronavirus disease 2019 (COVID-19). Multiple recent studies have investigated prone positioning in both non-mechanically ventilated and mechanically ventilated patients with COVID-19 [10,11,12].

This promising adult data prompted pediatric studies, including a randomized control trial demonstrating that prone positioning could be done safely in pediatric patients [13] and improves oxygenation [14]. Furthermore, the pediatric acute lung injury and consensus conference (PALICC) recommended to consider it in severe pediatric acute respiratory distress syndrome (PARDS) [15].

Manually prone positioning a patient can be challenging, particularly in larger patients. The number of providers, physical strength, and ability to routinely turn the patient can all complicate the care. In adults, the use of a rotational bed (RotoProne™) has eased the care of these patients and expanded the use of this therapy to patients who were previously not able to receive it [16,17]. As there appears to be little published data, the use of a rotational bed in children is unique. Therefore, we sought to describe this novel use of a rotational bed in critically ill children at a large academic, quaternary care hospital. In this report, we describe the practice patterns of utilizing a rotational bed, the pediatric patient population that was placed on a rotational bed, and report any complications that occurred with its use. We also describe oxygenation indices both prior to rotational bed therapy and while on rotational bed therapy.

## 2. Materials and Methods

A single center, retrospective case series was performed with patients who utilized a rotational bed as an adjunctive therapy for PARDS between 1st January 2016 through 31st December 2019. The study was designed to be a feasibility study on the utilization of rotational bed therapy in pediatric patients. The study was conducted in accordance with the Declaration of Helsinki and the protocol was approved by the Indiana University Institutional Review Board on 01/03/2019, protocol number 1812833300. Inclusion criteria were age equal to or less than 18 years, diagnosis of PARDS by PALICC criteria, and use of a rotational bed.

At our institution, placing a patient on the RotoProne™ bed requires a coordinated effort with one respiratory therapist (RT), two to three nurses, and careful monitoring. After transitioning the patient to the rotational bed in the supine position, safety checks and preparations are made. First, the endotracheal tube (ETT) is secured using tape instead of the endotracheal tube attachment device (ETAD). This is done for safety reasons; the ETT is more secure with tape as oral secretions may loosen the ETAD sticker point and allow the tube to slide through the holder. The ventilator tubing is placed in the equipment support device at the head of the bed. The patient’s skin is padded with allyvn or a padded barrier to prevent pressure ulcers. Intravenous lines are directed out of the head of the bed in the support device and the foley catheter and/or chest tubes, if applicable, are routed through the foot of the bed. After the preparatory work is completed, the patient is padded and strapped into the bed securely. While turning the patient prone, the RT monitors the ETT from the head of the bed. One nurse, also at the head of the bed, monitors equipment and another nurse controls the rotation from the foot of the bed. After the patient is prone, they are preferably placed in reverse Trendelenburg of −11 degrees and rotated right to left at 20–30 degrees. Final safety checks ensue with observation of how the patient’s positioning appears in the bed, making sure the lines and tubes are secure and placing an absorbent pad below the patient’s head to absorb any pooled secretions. Certain nurses have had special training in the use of rotational beds and function as “superuser’s” who help ensure safe utilization of this bed. It is important to note that rotational therapy beds were initially developed for use in adult patients but can be utilized in pediatric patients. There are no current pediatric specific rotational therapy beds, therefore it is important to use care when placing pediatric patients on these beds. At our institution, prone positioning is done at the clinician’s discretion. Currently, there is not a protocolized way that patients are placed on rotational beds. There is also no protocol for how long the patient should remain prone or how frequently they should be rotated back to supine. While the length of time the patient is left prone is at the clinician’s discretion, at our institution, patients are commonly placed prone for 16 h and supine for 8 h. 

Data collection included patient demographics, diagnoses, severity of illness, and critical care interventions and durations. PARDS specific variables were collected including mechanical ventilation strategies, adjunctive therapy utilization, fluid balance, and baseline oxygenation index (OI) and oxygenation saturation index (OSI) scores. If only OSI was available, the data point was transformed into OI equivalents using the equation OSI = 2.76 + 0.547 × oxygenation index. We collected rotational bed specific variables including duration of prone positioning and complications or adverse events. Sequential OI equivalent scores were collected every six hours from initiation of the rotational bed through the first 48 h of use. Lastly, we collected outcome related variables including mortality, hospital length of stay (LOS), length of mechanical ventilation, PICU LOS, and disposition. The data were obtained by review of the electronic medical record. All data were recorded and stored in a secure database on REDCap. RStudio (version 3.6.1) was used to perform statistical analysis [18]. Simple descriptive statistics, such as medians, frequencies, and percentages, were reported. Tests for normality were done utilizing Shapiro-Wilk tests. OI equivalents at each 6 h epoch were compared to the OI prior to initiation of the rotational bed using Wilcoxon signed-ranked tests. 

## 3. Results

**Figure 1:**
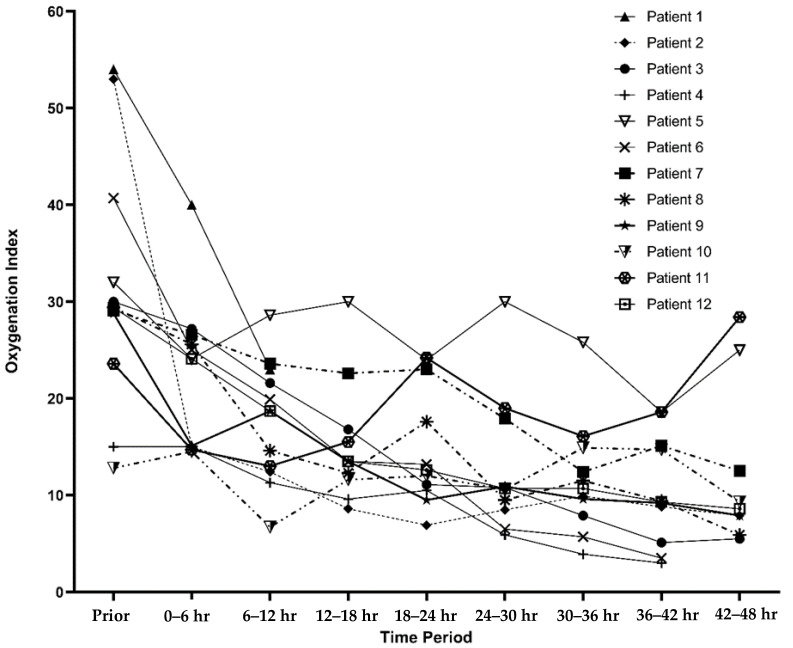
Oxygenation Index trends while proned displayed by individual patient. Figure Legend: Demonstration of oxygenation index trends prior to prone positioning and then at 6 h time epochs up to 48 h. If an oxygenation index was not available, then the oxygenation saturation index was back transformed into oxygenation index as was done by Khemani et al. [19]. Each line represents a different patient in this case series.

During this study period, 12 children with PARDS were placed on a rotational bed. Ages ranged from 12 to 17 years. Eight (67%) of the patients were male. Heights ranged from 152 to 170 cm and weights ranged from 52–120 kg. A summary of patient characteristics is compiled in Table 1. The patients had a median PELOD-2 score of 15.5 (IQR: 11, 21) prior to initiation of a rotational bed. Two patients had moderate ARDS, while 10 had severe ARDS with a median OI of 29.7 (IQR: 27.5, 36.4) prior to prone positioning. The oxygenation index trends for each patient are described in Figure 1. 

**Figure 2:**
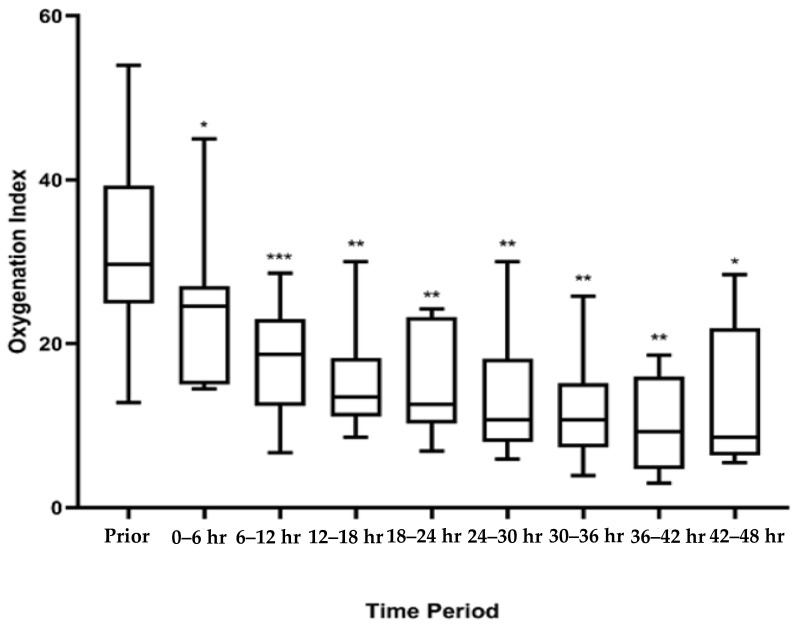
Oxygenation Index Trends After Starting Rotational Bed Therapy. Figure Legend: Comparison of oxygenation index trends prior to prone positioning and then after at 6 h intervals for the first 48 h. After prone positioning oxygenation indices improved over time. Oxygenation indices reported as medians, IQR, and ranges for each period examined. * = *p*-value < 0.05, ** = *p*-value < 0.01, *** = *p*-value < 0.001.

Patients were on the rotational bed for a median of 65.5 h (IQR: 41, 89.7). One patient had worsening lung compliance and prohibitive peak inspiratory pressures requiring discontinuing rotational bed therapy after 4 h. None of the patients had any central line dislodgements, severe facial edema, pressure injuries, unplanned extubations, endotracheal tube occlusions, or falls. Three (25%) of the patients had arterial lines malfunction while on the rotational bed necessitating replacement of arterial access. From the documentation in the medical records, it was unclear what the etiology of the malfunctions were. There was a statistically significant decrease in OI after initiation of prone positioning (Figure 2).

Multiple adjunctive therapies were given to the patients in this case series. Table 2 describes the therapies given to each patient. High frequency oscillatory ventilation (HFOV) was utilized in 50% of the patients. Airway pressure release ventilation was utilized in one of the patients. All patients received neuromuscular blockade for a median duration of 5.5 days (IQR:3.6, 7.7). Inhaled nitric oxide was utilized in 50% of the patients. Corticosteroids were administered in 50% of the patients for treatment of PARDS. None of the patients were placed on extracorporeal life support. The adjunctive therapies used were not standardized and were dependent on PARDS severity and physician’s preference. 

Table 3 describes length of stay data, length of mechanical ventilation, fluid overload status, vasoactive utilization, and renal replacement therapy utilization. Patients were mechanically ventilated for a median of 16 days (IQR: 13, 31). Tracheostomy tube was required in three (25%) of the patients who were discharged home on chronic respiratory support. The median length of hospital stay was 32 days (IQR: 21, 40). The median ICU length of stay was 26.5 days (IQR: 15.8, 32.3). Vasoactive infusions were used in eight (67%) patients and one patient required renal replacement therapy. One patient died in the cohort, secondary to progression of multiple organ dysfunction syndrome with subsequent cardiac arrest. We do not believe that this mortality was in any manner related to the utilization of rotational bed therapy in this patient. 

## 4. Discussion

This case series demonstrates that the use of a rotational bed can be done safely in children. To our knowledge, this is the first study to describe rotational bed utilization in the pediatric population. There were no serious adverse events thought to be secondary to the rotational bed. Three patients had a malfunction of their arterial lines while on the rotational bed. Unfortunately, we could not ascertain the cause of malfunction, but it will be imperative to closely monitor arterial lines while these patients are placed on rotational beds. While it is unfortunate that arterial lines were lost, it is important to note that no unplanned extubations or endotracheal tube obstruction occurred.

Data from adults with acute respiratory distress syndrome have demonstrated improved oxygenation and survival with prone positioning. The data are so compelling that prone positioning is considered a front-line therapy for adults with ARDS [9,20]. The benefit seen in adult patients led to a recommendation from the Pediatric Acute lung injury consensus conference to consider prone positioning in severe PARDS [15]. However, it appears that prone positioning may be under-utilized in PARDS for children treated in the United States [21]. This low frequency of prone positioning seen in this international study may be secondary to a negative randomized trial for prone positioning in pediatrics [14].

Despite this negative trial, prone positioning likely has a role in a subset of patients with PARDS, particularly with the consensus statement recommending its consideration for severe PARDS and recommending further pediatric study on stratification for severity [22]. Prone positioning has been shown in multiple studies to improve intrapulmonary shunt fraction in injured lungs, which leads to improvement in oxygenation [23,24,25]. Multiple observational pediatric studies have also demonstrated improvement in oxygenation [26,27]. Similarly, in this small case series, there was a statistically significant improvement in oxygenation indices after initiation of a rotational bed. To account for missing oxygenation index data in patients without arterial blood gas data, we performed back-transformation of oxygenation saturation indices. This was done in a similar manner to what has been done in other studies using the equation OSI = 2.76 + 0.547 × oxygenation index [19,28]. In our study, the patients had statistically significant improvement in oxygenation after prone positioning. Our small case series adds more evidence to the beneficial oxygenation effects seen when patients are positioned prone. While this study is too small to recommend the use of rotational therapy beds in all children with PARDS, it does suggest that in our small cohort, rotational bed therapy did not result in worsening pulmonary outcomes and perhaps had a beneficial effect on oxygenation. There are ongoing efforts to better understand the utility of prone positioning in PARDS (PROSPECT trial, NCT03896763), but this study did not specifically evaluate the use of a rotational therapy bed. Future prospective multi-center studies investigating the use of a rotational bed while controlling for important factors such as severity of illness would be important to assure generalizable safety, and to better ascertain the role of physician bias.

The various sizes of patients encountered in the PICU can make a standardized approach to prone positioning difficult. Larger patients can be more challenging to place prone. Therefore, a rotational bed may assist with this challenge. Rotational therapy beds have multiple benefits over manual prone positioning. First, the labor involved to reposition these patients once they are on the rotational therapy bed is much less intensive. Second, repositioning can be done with the bedside nurse alone instead of the multiple providers required to manually reposition them. Third, patients on rotational therapy beds are able to rotate laterally to each side, which has been demonstrated to reduce risk of ICU-acquired infections in the adult population [29]. Here, we demonstrate that in an older pediatric population, rotational therapy beds can be used safely with a multidisciplinary approach to placing the patient on the bed and monitoring its use carefully. 

This report has some limitations. Given its retrospective nature and small sample size, it is difficult to describe rare occurrences and complications that could be encountered with rotational bed therapy. Given it was only done at a single center, our results may not be generalizable to all settings, especially in resource limited settings where access to these specialty beds may be limited. While there was a statistically significant improvement in oxygenation over time, this was not compared to a control group and therefore no conclusions can be inferred about the benefit of prone positioning compared to manual prone positioning or supine positioning. The role of adjunctive therapies was also not controlled for and may have a significant role in perceived oxygenation benefit.

## 5. Conclusions

Rotational beds can be safely utilized in pediatric patients. It may be considered as a tool to aid in prone positioning for larger pediatric patients with PARDS where it would be more difficult to manually prone.

## Figures and Tables

**Table 1:** Patient demographics for each subject included in the case series.

Patient	Diagnosis	Age	Sex	Race/Ethnicity	Admission Weight	BMI	Admission PRISM	Admission PELOD
1	Pancreatitis	12	M	Caucasian	71.5	33.6	4	12
2	Aspiration Pneumonia	17	F	Caucasian	120	48.2	6	21
3	Aspiration Pneumonia	14	F	Caucasian	46.7	22.2	15	12
4	Aspiration Pneumonia	17	M	African American	50.3	19.7	10	21
5	Bacterial Pneumonia	16	M	African American	110	31.1	2	1
6	Aspiration Pneumonia	14	M	African American	53.9	18.6	8	11
7	Bacterial Pneumonia	12	M	Caucasian	105	42.1	11	11
8	Bacterial Pneumonia	14	F	Caucasian	60.8	26.4	20	11
9	Bacterial Pneumonia	10	M	Caucasian	56.7	22.9	6	10
10	Inhalation Injury	15	M	Caucasian	106	37.6	16	20
11	Eosinophilic Pneumonitis	17	M	Caucasian	52	19.7	5	11
12 *	Viral Pneumonia	14	F	Hispanic	55.3	20.7	12	22

Each number denotes a specific patient and is consistent throughout the tables. Age reported in years; Sex: M = male, F = female; Admit weight is listed in Kilograms; PRISM = Pediatric risk of mortality III score calculated at admission; PELOD = Pediatric logistic organ dysfunction-2 score calculated at admission; * = deceased.

**Table 2:** Ancillary Therapy utilized for each subject in case series.

Patient	Prone Duration	NMB Duration	HFOV	APRV	iNO Use	Corticosteroid Use
1	0.6	7.3	Yes	No	No	Yes
2	4.8	7.9	Yes	No	Yes	Yes
3	2.1	2.3	No	No	Yes	No
4	0.7	1.8	No	No	Yes	Yes
5	4.5	4.1	No	No	No	No
6	1.8	11.8	Yes	Yes	No	No
7	2.7	6.8	No	No	Yes	Yes
8	2.8	3.3	Yes	No	No	No
9	0.1	3.8	Yes	No	Yes	Yes
10	3.5	7.5	Yes	No	Yes	No
11	2.7	4.2	No	No	No	Yes
12 ^	4.9	8.1	No	No	No	Yes

Time Prone is listed in days; NMB duration = duration of neuromuscular blockade in days; HFOV = high frequency oscillatory ventilation; APRV = airway pressure release ventilation; Ino = inhaled nitric oxide; Steroid use = those who were treated with corticosteroids for ARDS while on rotational therapy bed. ^ = deceased.

**Table 3:** Hospital data for each subject included in the case series.

Patient	Hospital LOS	ICU LOS	Length of IMV	Fluid Overload	Vasoactive Use	RRT Utilization
1	94	43	93 *	22%	Yes	Yes
2	21	19	14.6	0%	Yes	No
3	21	16	14.0	0.2%	Yes	No
4	51	29	12.2	8.2%	No	No
5	36	33	32 *	1.1%	No	No
6	30	24	17.5	10.3%	Yes	No
7	38	33	38 *	1.8%	Yes	No
8	46	41	22.4	7%	Yes	No
9	11	7	5.8	5.9%	Yes	No
10	21	15	13.7	0.2%	No	No
11	32	8	5.7	7.2%	No	No
12 ^	32	30	30.0	6.7%	Yes	No

Hospital LOS = hospital length of stay reported in days. ICU LOS = intensive care unit length of stay reported in days. Length of IMV = days of invasive mechanical ventilation. Fluid overload = percent fluid overloaded at initiation of prone positioning. Vasoactive use included epinephrine, norepinephrine, vasopressin, dopamine, and milrinone. RRT = renal replacement therapy. * = patients who underwent tracheostomy. ^ = deceased.
